# A proteomic insight into vitellogenesis during tick ovary maturation

**DOI:** 10.1038/s41598-018-23090-2

**Published:** 2018-03-16

**Authors:** Marina Amaral Xavier, Lucas Tirloni, Antônio F. M. Pinto, Jolene K. Diedrich, John R. Yates, Albert Mulenga, Carlos Logullo, Itabajara da Silva Vaz, Adriana Seixas, Carlos Termignoni

**Affiliations:** 1 Centro de Biotecnologia, Universidade Federal do Rio Grande do Sul, Porto Alegre, RS Brazil; 2 Department of Veterinary Pathobiology, Texas A&M University, College Station, TX USA; 3 Department of Chemical Physiology, The Scripps Research Institute, La Jolla, CA USA; 4 Laboratório de Sanidade Animal, Laboratório de Química e Função de Proteínas e Peptídeos and Unidade de Experimentação Animal, Universidade Estadual do Norte Fluminense Darcy Ribeiro, Campos dos Goytacazes, RJ Brazil; 5 Faculdade de Veterinária, Universidade Federal do Rio Grande do Sul, Porto Alegre, RS Brazil; 6 Departamento de Farmacociências, Universidade Federal de Ciências da Saúde de Porto Alegre, Porto Alegre, RS Brazil; 7 Departamento de Bioquímica, Universidade Federal do Rio Grande do Sul, Porto Alegre, RS Brazil

## Abstract

Ticks are arthropod ectoparasites of importance for public and veterinary health. The understanding of tick oogenesis and embryogenesis could contribute to the development of novel control methods. However, to date, studies on the temporal dynamics of proteins during ovary development were not reported. In the present study we followed protein profile during ovary maturation. Proteomic analysis of ovary extracts was performed by liquid chromatography-tandem mass spectrometry (LC-MS/MS) using shotgun strategy, in addition to dimethyl labelling-based protein quantification. A total of 3,756 proteins were identified, which were functionally annotated into 30 categories. Circa 80% of the annotated proteins belong to categories related to basal metabolism, such as protein synthesis and modification machineries, nuclear regulation, cytoskeleton, proteasome machinery, transcriptional machinery, energetic metabolism, extracellular matrix/cell adhesion, immunity, oxidation/detoxification metabolism, signal transduction, and storage. The abundance of selected proteins involved in yolk uptake and degradation, as well as vitellin accumulation during ovary maturation, was assessed using dimethyl-labelling quantification. In conclusion, proteins identified in this study provide a framework for future studies to elucidate tick development and validate candidate targets for novel control methods.

## Introduction

Ticks are hematophagous ectoparasites of importance for human and veterinary health, due to direct effects of the parasitism, and the pathogenic agents they can transmit^1^. During development, ticks go through four stages of life: egg, larva, nymph and adult. Members of the family Ixodidae undergo either one-host, two-host or three-host life cycles. Particularly, *Rhipicephalus microplus*, a major cattle parasite^2^ is a one-host tick; it remains on the same host throughout larval, nymph and adult stages, only leaving the host prior to laying eggs^3^.

*R. microplus* attaches to the cattle host as unfed larvae, and then proceeds to feed and moult through nymphal and immature adult stages, during a period of about 12 days. After mating, adult females start the slow feeding phase, when they swallow moderate amounts of blood for about 6 days^3^. The rapid engorgement phase takes place on the last 3 days on the host, when most of the blood is ingested. After completing the blood meal, the fully engorged females detach from the host and perform oviposition on the soil^4^.

Oogenesis is the development of female sex cells (oocytes) in the ovaries, while vitellogenesis is the process whereby ovarian and extraovarian tissues produce protein precursors and other molecules which are transported to and accumulated inside the oocytes^5^. In Ixodidea ticks, vitellogenin (Vg) is synthesised as a high molecular mass-precursor in fat body, gut and ovaries^6–9^. Vg is released into the haemolymph to be taken up by oocytes via a receptor mediated endocytosis^10^, and then accumulates inside the oocytes yolk granules, being thereafter named vitellin (Vt). Together with other yolk components, Vt is a source of nutrients for embryo development^11^.

Biological function depends on the interplay of gene and protein regulation, influenced by extracellular factors. Therefore, a comprehensive understanding of the profile of gene expression and the proteins present throughout ovary development is necessary to elucidate the events occurring during arthropod development. Transcriptomic profile analysis has been widely used in insect research^12^, and the gene expression profiles of several insect embryos during the early developmental stages have been characterised^13,14^. Nevertheless, there are few proteomic studies addressing the molecular basis of developmental regulation in arthropods. Moreover, despite its importance, tick ovary has not been studied in detail and, unfortunately, there is a paucity of reports showing the successive alterations in protein profile during tick ovary maturation. In this sense, various aspects of *R. microplus* ovary maturation have been addressed at the level of individual genes and/or proteins, which is insufficient to uncover the underlying mechanisms of ovary development^15^. In arthropods, comparative proteomic approaches were applied to analyse reproduction strategies used by the parasitoid wasp *Venturia canescens*^16^, to compare activated and inactivated ovaries from workers bees *Apis mellifera*^17^, and different ovary stages in the shrimp *Metapenaeus ensis*^18^, and also to study differences in ovaries of domestic and wild shrimps *Penaeus monodon*^19^.

The study of *R. microplus* embryogenesis^20^ can contribute to a better understanding of embryo metabolism and development in ticks, as well as in other arthropods. Despite the importance of this parasite, no proteomic study on ovary development of *R. microplus* has been reported so far. Until now, there are only two comparative studies addressing tick ovary using proteomics^21^ or transcriptomics^22^, but these studies are focused on *Babesia bovis* infection. In the present study, proteomic profiles were analysed in whole tissue extracts of tick ovary representing sequential stages of ovary maturation. Samples were analysed by liquid chromatography-tandem mass spectrometry (LC-MS/MS) based on shotgun strategy. In addition, dimethyl labelling-based protein quantification was used to identify differences in protein variation. In summary, the results shown here provide a clearer view on the post-transcriptional events in *R. microplus* ovary maturation, pointing out protein modulation during ovary development. This work provides a better understanding of how tick ovary develops, and how protein synthesis is regulated during embryogenesis. Consequently, this knowledge offers opportunities for advancing novel targets of tick control methods, since the inhibition of protein function may result in a decreased viability of the eggs, or even in premature mortality.

## Results and Discussion

### Ovary development

Oocyte formation is the initial step in embryonic development and, like ovary maturation, is heavily affected by blood ingestion and consequently by female weight. Balashov^23^ proposed a categorisation of tick oogenesis according to individual oocyte development. However, oocyte development is asynchronous from the beginning of yolk uptake, and it is possible to find all Balashov-oocyte stages occurring at the same time during ovarian maturation^24^. Thus, using *Amblyomma hebraeum* tick as a model, Seixas *et al*.^25^ proposed a new classification that comprises the degree of oocyte development and the size of the ovary. According to this classification, ovarian growth phase (OGP) 1 is characterised by a thin and translucent ovary with visible oocyte nuclei. In OGP 2, ovaries are longer and thicker than in OGP 1, and oocytes are opaque with no visible nuclei, while in OGP 3 many oocytes acquire a brown colour due to yolk granules accumulation. More like a pre-ovulation stage, in OGP 4 large and yolk-filled oocytes prevail, and yolk spheres are visible. In OGP 5, when in *A. hebraeum* oviposition begins, the ovary appearance is the same as in OGP 4, but ovulated oocytes are visible in the ovary lumen^25^. It is important to note that *A. hebraeum* females take 10 days after detachment from the host to start oviposition. In contrast, in *R. microplus* females this period takes only 3 days. Nevertheless, the OGP system classification is useful to normalise ovary maturation period, thus allowing to compare protein levels among female ovaries.

### Tick ovary proteome overview

**Figure 1:**
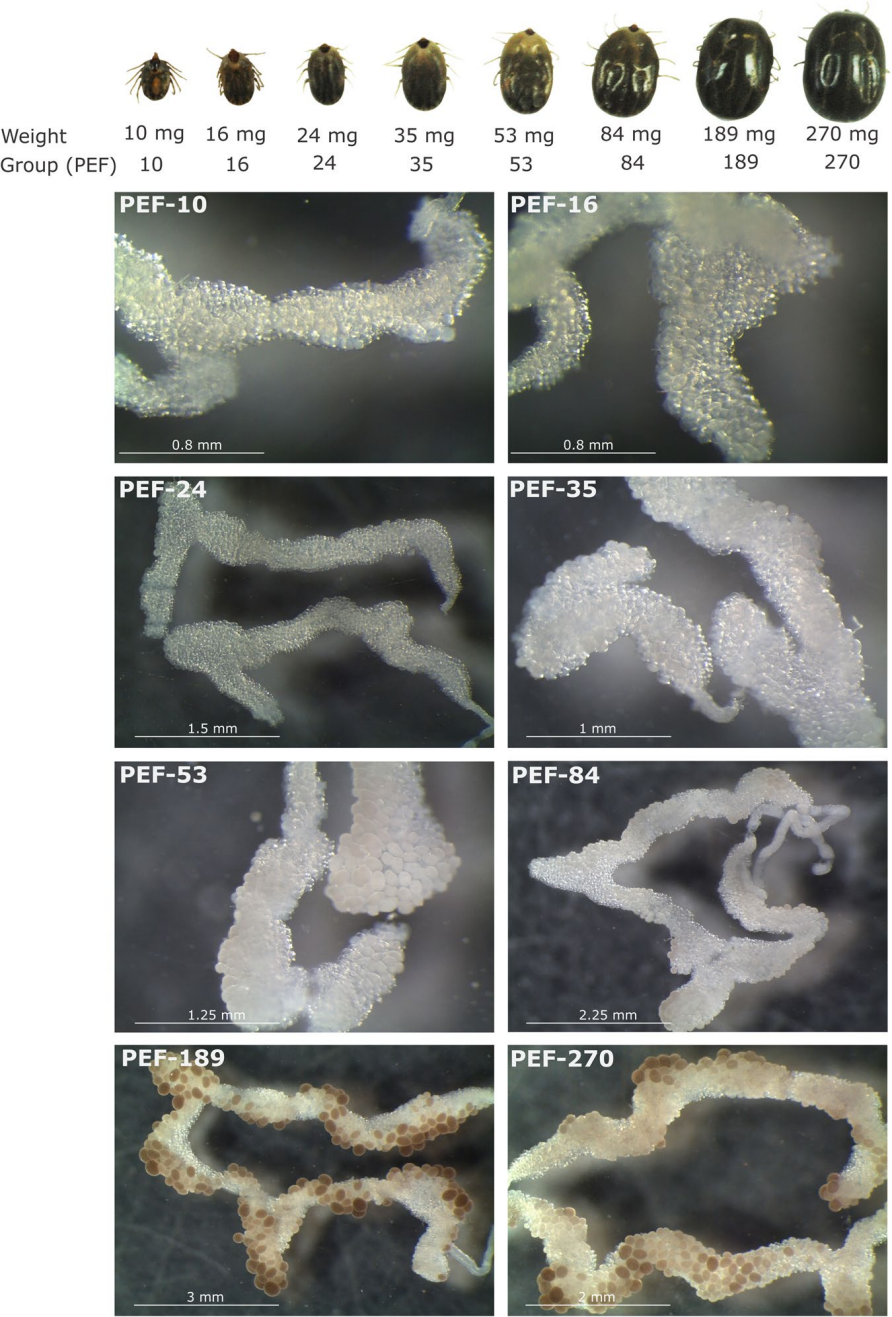
Representative images of specimens and ovaries of *Rhipicephalus microplus* partially engorged females (PEF) groups. PEF were grouped according to average weight of individuals (10 ± 1.73 mg, 16 ± 1.22 mg, 24 ± 1.90 mg, 35 ± 2.84 mg, 53 ± 2.49 mg, 84 ± 8.36 mg, 189 ± 17.17 mg, 270 ± 16.71 mg). Ovary images (from PEF-10 to PEF-270) exemplify the ovaries from PEF groups used in this study, which correspond to ovarian growth phase (OGP) categorisation^25^: PEF-10, -16 and -24 (OGP 1); PEF-35 (OGP 2); PEF-53 and -84 (OGP 3); PEF-189 and -270 (OGP 4). Numbers of ticks composing each group are: PEF-10 (n = 10), PEF-16 (n = 9), PEF-24 (n = 28), PEF-35 (n = 23), PEF-53 (n = 8), PEF-84 (n = 9), PEF-189 (n = 5), PEF-270 (n = 5). FEF groups D1, D2, D3, and D4 were composed by ovaries from 5 ticks.

**Figure 2:**
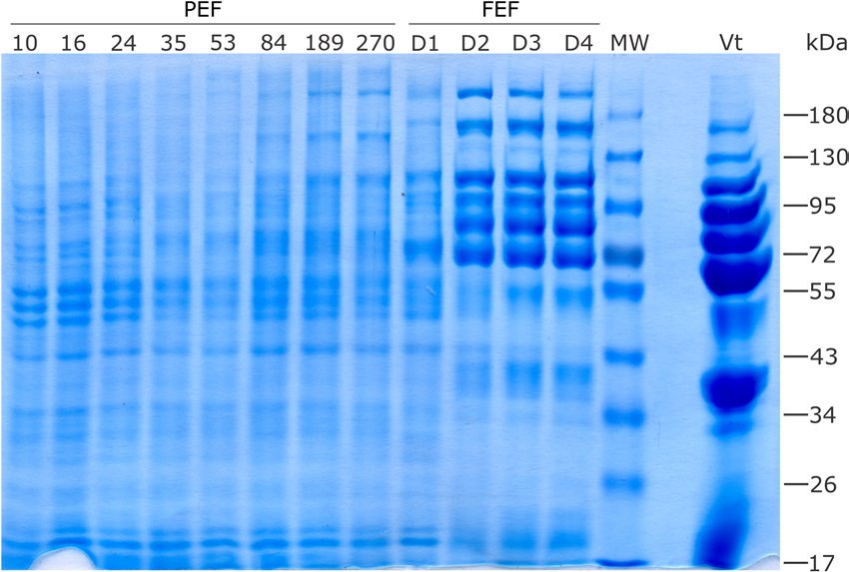
Electrophoretic profile of *Rhipicephalus microplus* ovary proteins. The protein profile of ovaries from partially engorged female groups (PEF-10, -16, -24, -35, -53, -84, -189, and -270) and fully engorged females (FEF) groups, which were categorised according to post-detachment day (D1, D2, D3 and D4), were analysed by SDS-PAGE. Approximately 50 µg of total protein was resolved on 10% gels and stained with Coomassie blue. MW: molecular weight standards, indicated in kDa on the right; Vt: purified *Rhipicephalus microplus* vitellin. Numbers of ticks composing each group are: PEF-10 (n = 10), PEF-16 (n = 9), PEF-24 (n = 28), PEF-35 (n = 23), PEF-53 (n = 8), PEF-84 (n = 9), PEF-189 (n = 5), PEF-270 (n = 5). FEF groups D1, D2, D3, and D4 were composed by ovaries from 5 ticks. This image is representative of two independent SDS-PAGE experiments.

In this work, ovaries from *R. microplus* partially engorged females (PEF) and fully engorged females (FEF), which were manually collected, were analysed to perform a temporal proteomic study. PEF were divided in eight groups according to average weight of individuals (as an indicative of blood meal acquisition and ovary maturation) ranging between 10 and 270 mg (groups PEF-10 to PEF-270) (Fig. 1). FEF were grouped according to days post-detachment (FEF-D1 to FEF-D4) (details in section “Ovary extracts and protein digestion” in Materials and Methods). These groups represent sequential stages of ovary maturation and oogenesis. The electrophoretic profiles of PEF and FEF ovary proteins reveal a clear difference in the amount of some proteins (Fig. 2). As ovaries mature, vitellin (Vt) accumulation (polypeptides ≥55 kDa) is visible, especially in late vitellogenesis (after PEF-53) and in FEF groups. Also, the profiles show that the number of polypeptides ≤55 kDa decreases as ovary develops. These electrophoretic patterns correlate with microscopy observations (Fig. 1), where ovaries go from off-white to a brownish colour, due to the amount of Vt-containing yolk granules (Vt has a typical colour due to its haeme content). To contemplate a whole panel of changes in the protein profile observed during ovary development until the end of oogenesis, experimental groups PEF-10, -24, -35, -53, -84, -189, and -270, and FEF-D1 and -D3 were subjected to proteomics analyses (Fig. 2).

**Figure 3:**
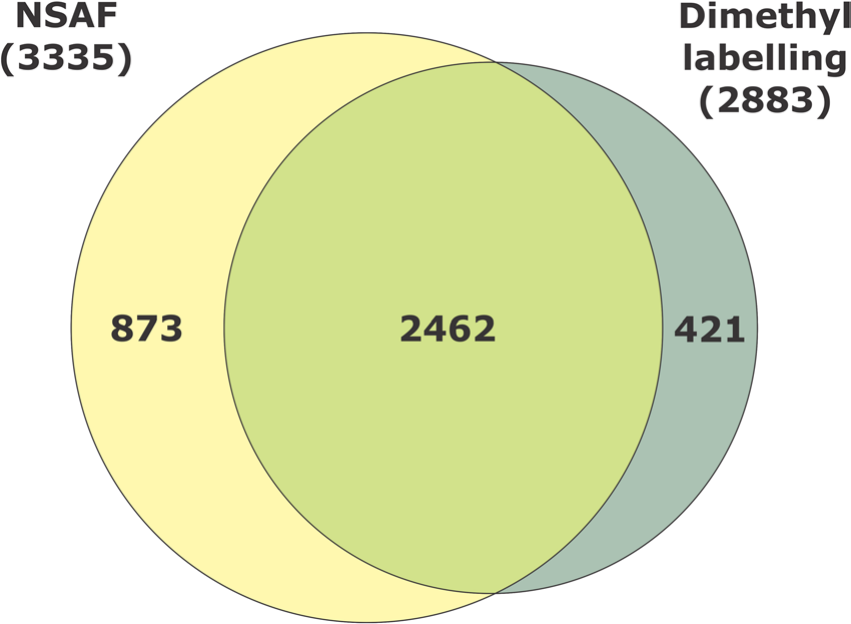
Number of proteins identified in *Rhipicephalus microplus* ovaries using two different approaches. Venn diagram comparing number of proteins identified in semi-quantitative normalised spectral abundance factor (NSAF) analysis, and quantitative dimethyl labelling analysis.

**Figure 4:**
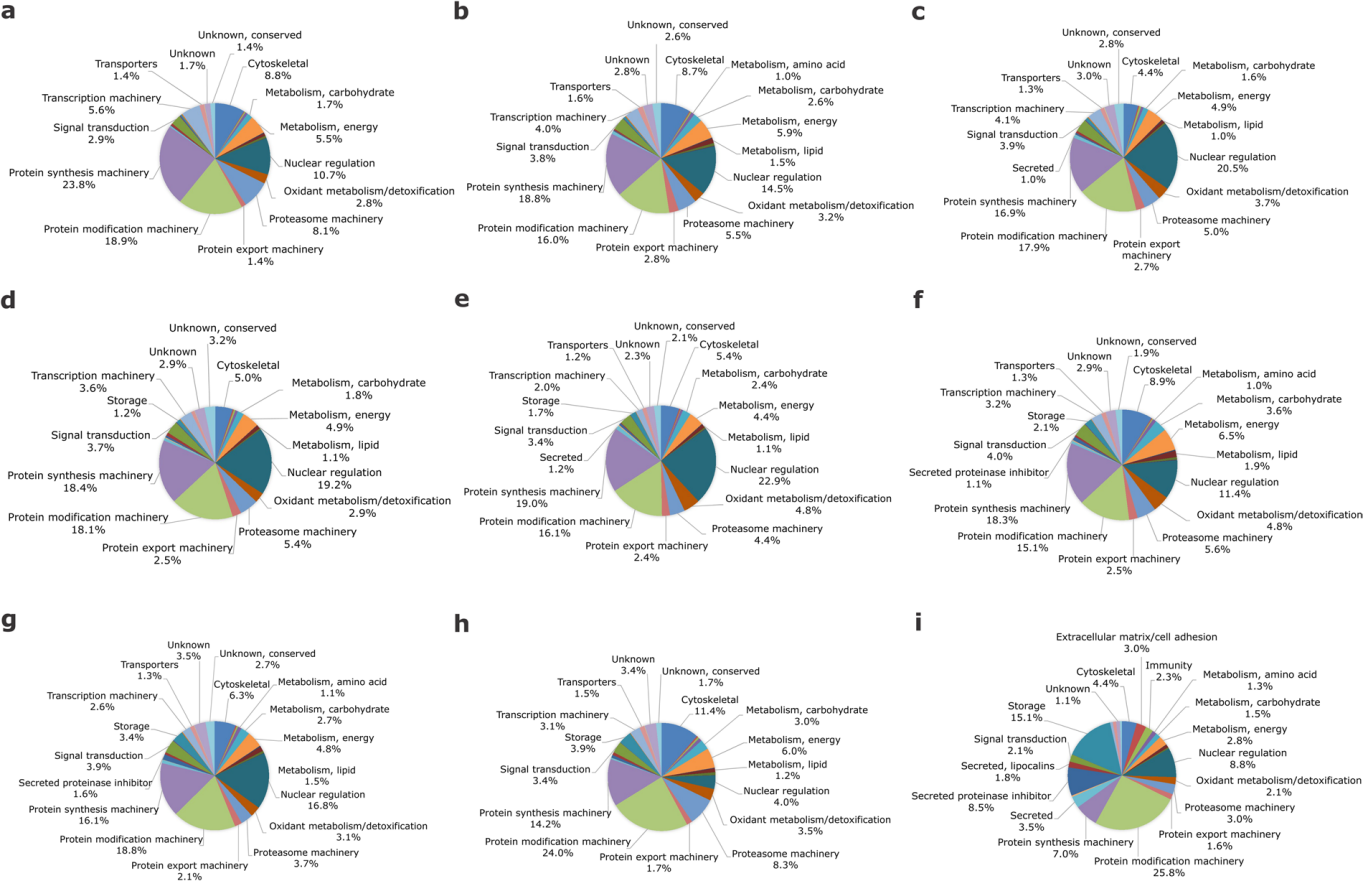
Functional clusterisation of *Rhipicephalus microplus* ovary proteins. Proteomic analysis was performed in partially engorged females, groups PEF-10 (**a**), -24 (**b**), -35 (**c**), -53 (**d**), -84 (**e**), -189 (**f**), and -270 (**g**); and fully engorged females, groups FEF-D1 (**h**) and -D3 (**i**). Pie charts represent the percentage of proteins found in each group respective to normalised spectral counting (NSAF) for each sample. Numbers of ticks composing each group are: PEF-10 (n = 10), PEF-16 (n = 9), PEF-24 (n = 28), PEF-35 (n = 23), PEF-53 (n = 8), PEF-84 (n = 9), PEF-189 (n = 5), PEF-270 (n = 5). FEF groups D1, D2, D3, and D4 were composed by ovaries from 5 ticks.

Altogether, a total of 3,756 unique proteins were found in the proteome of *R. microplus* ovaries. Among them, 3,335 proteins were identified using normalised spectral abundance factor (NSAF) semi-quantitative approach, while 2,883 proteins were identified by dimethyl labelling quantitative method. From the proteins detected by dimethyl labelling, there are 421 unique proteins when compared to NSAF. Thus, 2,462 proteins were detected in common by NSAF and dimethyl labelling (Fig. 3; see Supplementary Tables S1, S2 and S3). Dimethyl-labelled proteins with *p* value and ratio variance ≥0.06 were excluded from the analysis. Through NSAF semi-quantification, more than 2,000 proteins were identified in PEF-24, -35 and -270 groups; more than 1,000 in PEF-10, -53 and -189 and FEF-D1 groups; and more than 800 proteins in FEF-D3 group (Fig. 4 and Supplementary Table S2). Despite a distant phylogenetic relationship, the number of proteins described here in *R. microplus* is similar to what was reported in a comparative study of ovarian proteome performed with the shrimp *Penaeus monodon*, which found a total of 1,638 proteins^19^. Also similarly to the present study, Velentzas *et al*.^26^ reported the descriptive proteome of *Drosophila melanogaster* ovary, in which 2,103 proteins were found.

After mating, ovary engages in oogenesis to produce eggs that will provide embryo development; therefore, this organ is in constant physiological and biochemical modification. Broadly, in all ovary stages circa 80% of the proteins are involved in basal metabolism. These proteins are clustered as: synthesis and modification machineries, nuclear regulation, cytoskeletal proteins, proteasome machinery, transcription machinery, energetic metabolism, extracellular matrix/cell adhesion, immunity, oxidation/detoxification metabolism, signal transduction, and storage. Also, the NSAF overview highlights that protein amount in some clusters changes over time during the temporal analysis performed. Proteins related to transcriptional machinery, protein synthesis machinery, and nuclear regulation show a decrease in abundance as ovaries develop, while storage proteins are upregulated (Fig. 4).

**Figure 5:**
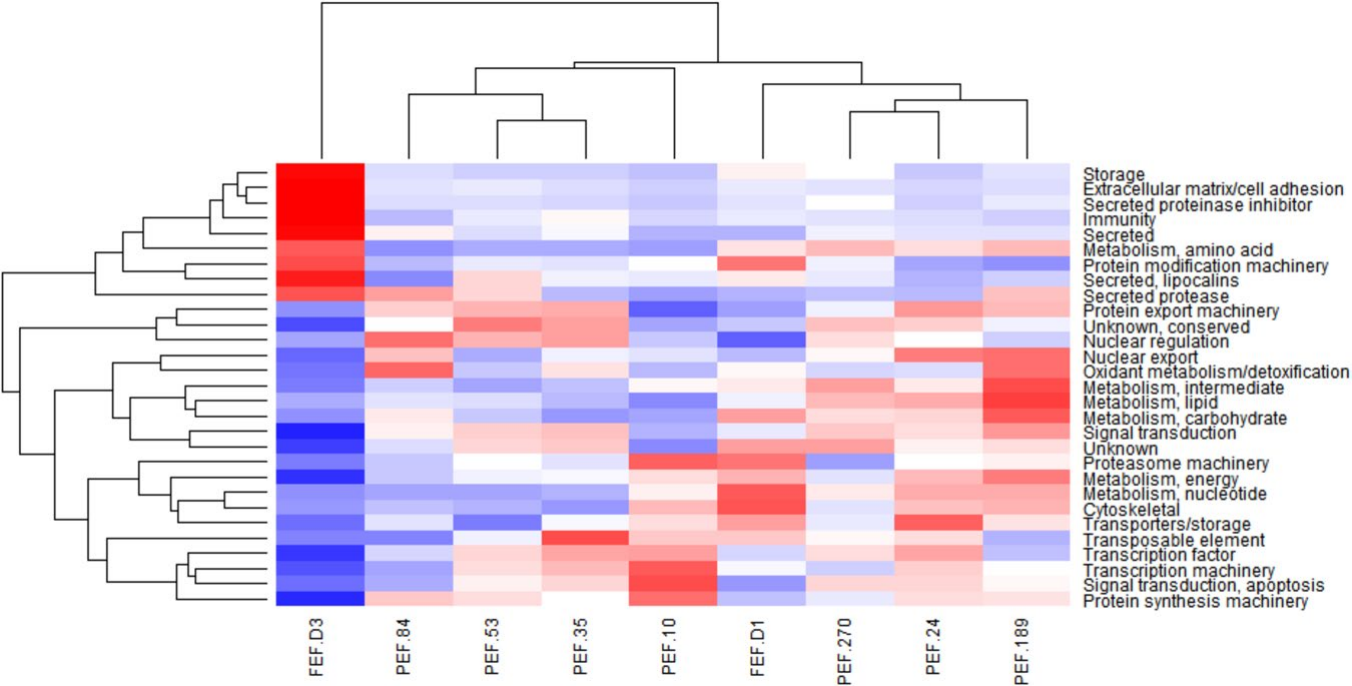
Protein abundance in *Rhipicephalus microplus* ovaries. Heat map of NSAF data for each protein is expressed as a per cent of total NSAF per group within each category. Z-scores were calculated and used to generate heat maps as described in Material and Methods section. Red colour indicates proteins of high abundance and blue colour indicates proteins of low abundance. Dendrogram on the left shows protein clustering according to functional annotation.

The temporal variation among protein levels was also supported by heat map analysis, which demonstrates the different patterns among ovary protein clusters (Fig. 5). The heat map shows that there are variations in protein profile, which are dependent of female weight, and allowed to identify a relationship between protein functions and ovary development. Clearly, this analysis reveals some trends: (i) groups representing early stages of ovarian development are mostly clustered in one branch, including PEF-10, PEF-35, PEF-53, and PEF-84; (ii) groups representing late stages of ovarian development (and group PEF-24) are clustered in another branch, including PEF-189, PEF-270 and FEF-D1; (iii) group FEF-D3 forms a separate branch. The abundance of proteins involved in storage, extracellular matrix, secreted proteins, protease inhibitors, immunity, amino acid metabolism, protein modification, lipocalins, and proteases increased significantly during ovary development, being upregulated at the end of ovary maturation (FEF-D3) (Fig. 5). Conversely, the abundance of proteins related to signalling, protein synthesis, and metabolism decreased over time (Fig. 5). Altogether (Figs 4 and 5), these differences in protein profile during ovary maturation indicate that when ticks detach from the host, ovaries are almost fully mature, with reduced synthesis of new proteins; at this point, the storage of elements important for embryo development becomes more conspicuous.

**Figure 6:**
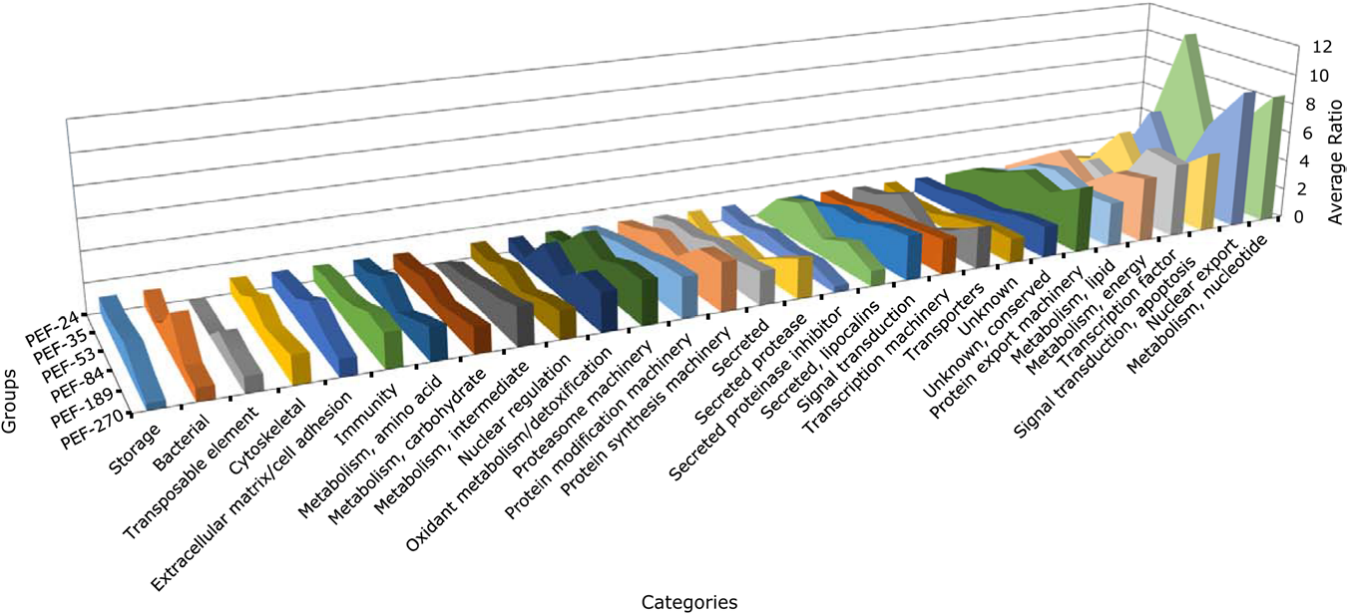
Quantitative proteomic analysis of *Rhipicephalus microplus* partially engorged females (PEF) ovaries. Dimethyl labelling quantification of ovary proteins in PEF groups -24, -35, -53, -84, -189 and -270 was performed. Area chart shows the variation in protein levels (y axis) as a function of ovary maturation (z axis). The x axis shows the categories in which proteins were clustered. The data from each group was normalised relative to the group PEF-24. Numbers of ticks composing each group are: PEF-10 (n = 10), PEF-16 (n = 9), PEF-24 (n = 28), PEF-35 (n = 23), PEF-53 (n = 8), PEF-84 (n = 9), PEF-189 (n = 5), PEF-270 (n = 5).

For more accurate protein quantification, a dimethyl labelling relative quantification was performed in the groups PEF-24, -35, -53, -84, -189 and -270. To analyse changes in ovary protein profile, the data of each group was normalised relative to the PEF-24 group, the smallest individuals who are in the beginning of the blood feeding. This allows to observe variations in protein amount among the developmental stage categories, i.e. during ovary maturation (Fig. 6). Proteins related to cell growth, differentiation, and development (nucleotide metabolism, nuclear export, transcription factor, signal transduction for apoptosis, energy metabolism, lipid metabolism, and protein export machinery) are the most abundant throughout ovary development. Moreover, proteins related to intermediate metabolism, lipocalins, and transposable elements are overall much less abundant, being absent in the beginning of ovary development (Fig. 6).

Altogether, the data corroborates previous observations from studies focusing on individual tick ovary proteins^15^. Because of their fundamental role in ovary maturation and embryo development, we selected proteins involved in vitellin processing and energetic metabolism to be analysed and discussed in detail.

### Vitellogenesis during ovary development

Vt, a lipoglicoprotein, is the main source of nutrients for embryo development. Its degradation depends on a developmentally regulated acidification of the yolk granules, starting immediately after oviposition. Until the time of egg hatching, 40% of Vt content is consumed^27^, implicating a very controlled enzymatic digestion mechanism^28^.

**Figure 7:**
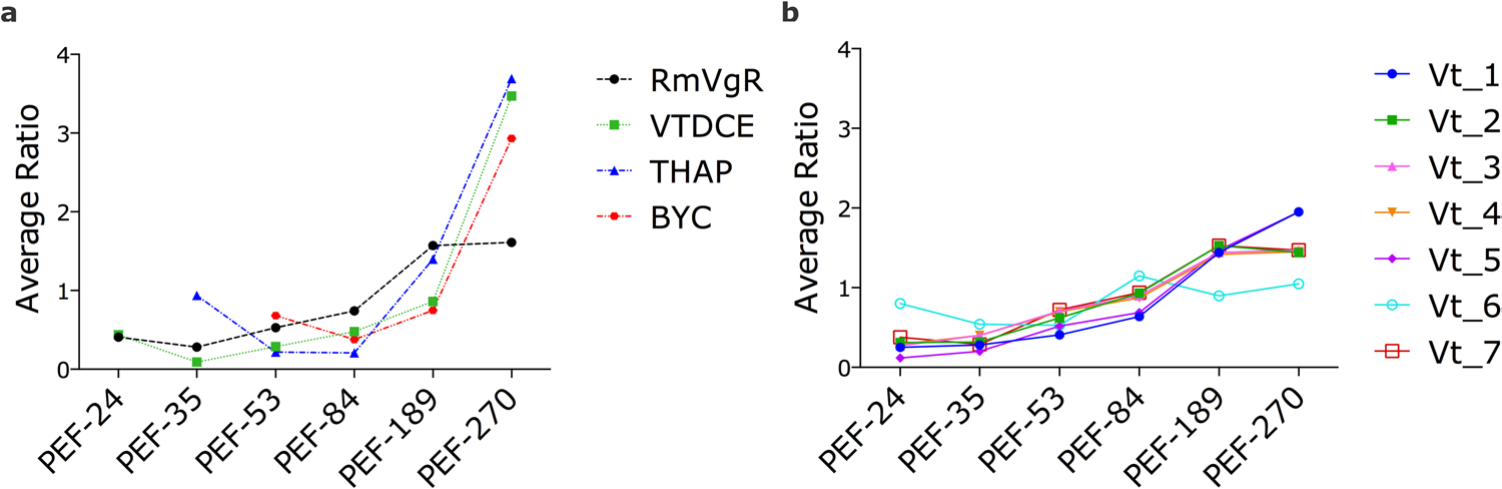
Profiles of abundance of proteins involved in yolk processing and vitellin (Vt) accumulation in *Rhipicephalus microplus* ovaries. Dimethyl labelling quantification was performed in ovary extracts of partially engorged female groups PEF-24, -35, -53, -84, 189, -270. For dimethyl labelling quantification, the ratio is the intensity detected for each peptide, relative to the internal standard (a protein pool of all samples used in this study). The average ratio of a protein is calculated as the mean ratio of its peptides. Chart (**a**) shows protein average ratio of the receptor involved in yolk uptake, *Rhipicephalus microplus* Vitellogenin Receptor (RmVgR), and the proteases implicated in yolk degradation, namely Vitellin-Degrading Cysteine Endopeptidase (VTDCE), Tick Haeme-binding Aspartic Proteinase (THAP), and *Boophilus* Yolk pro-Cathepsin (BYC). Chart (**b**) shows average ratio of Vt polypeptides. Numbers of ticks composing each group are: PEF-10 (n = 10), PEF-16 (n = 9), PEF-24 (n = 28), PEF-35 (n = 23), PEF-53 (n = 8), PEF-84 (n = 9), PEF-189 (n = 5), PEF-270 (n = 5).

**Figure 8:**
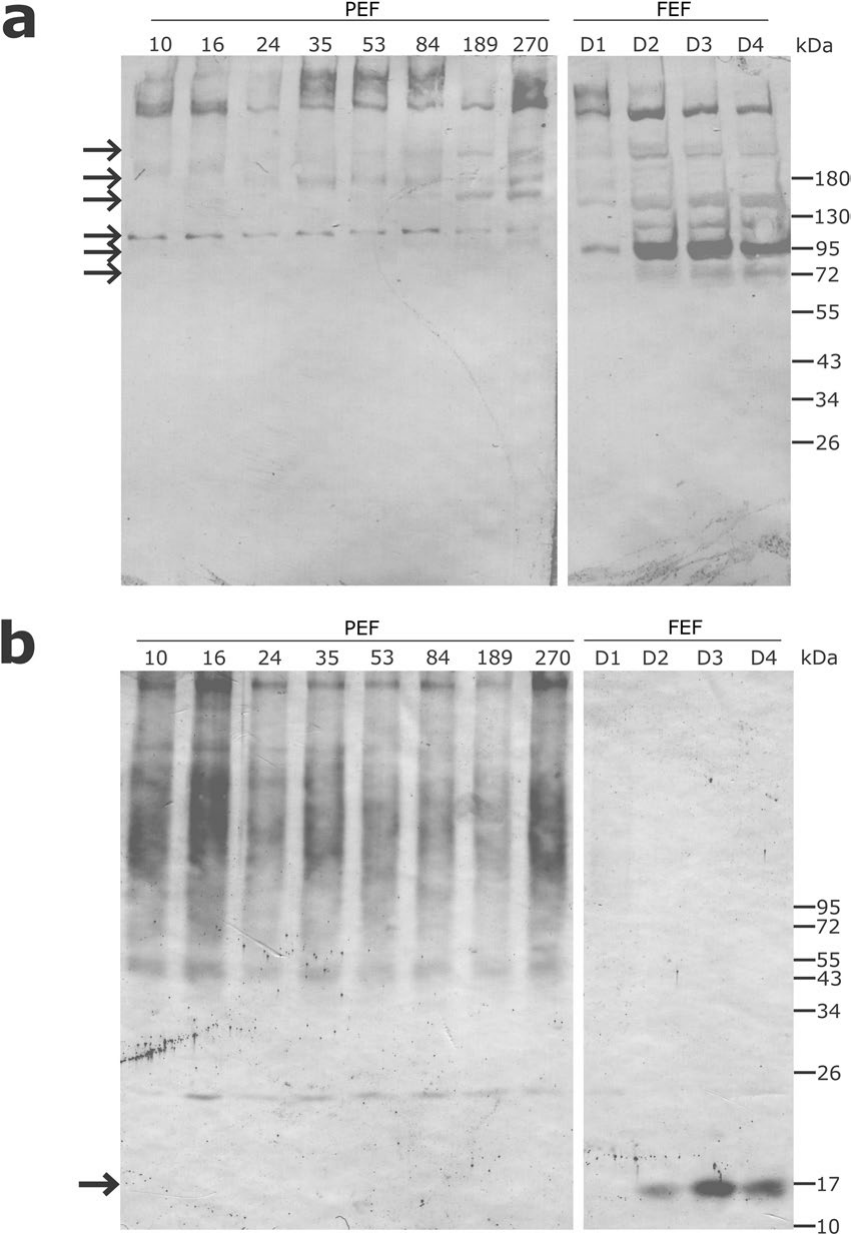
Vitellin (Vt) and vitellin degrading cysteine endopeptidase (VTDCE) in *Rhipicephalus microplus* ovaries analysed by western blot. Protein extracts from partially (PEF) and fully engorged females (FEF) ovaries were probed with rabbit antibodies (**a**) anti-Vt and (**b**) anti-VTDCE. PEF groups: -10, -16, -24, -35, -53, -84, -189, -270; FEF groups: -D1, -D2, -D3 and -D4. Gaps were created in the images to delineate the stages of ovary maturation, but both parts are from the same gel. This image is representative of two (for VTDCE) and four (for Vt) independent western blot. Numbers of ticks composing each group are: PEF-10 (n = 10), PEF-16 (n = 9), PEF-24 (n = 28), PEF-35 (n = 23), PEF-53 (n = 8), PEF-84 (n = 9), PEF-189 (n = 5), PEF-270 (n = 5). FEF groups D1, D2, D3, and D4 were composed by ovaries from 5 ticks. This image is representative of two (for VTDCE) and four (for Vt) independent western blot.

Therefore, to improve the understanding of the processes occurring during ovary maturation, critical proteases involved in yolk hydrolysis (BYC^29^, THAP^30^, VTDCE^31^), and a receptor responsible for Vg uptake by the oocytes, (*Rhipicephalus microplus* vitellogenin receptor, RmVgR)^32^, were selected for dimethyl labelling quantification and further analysis (Fig. 7a). These vitellin-degrading enzymes and RmVgR are present at high abundance during the rapid engorgement phase of PEF (PEF-189 and PEF-270). Additionally, seven Vt polypeptides were identified (Figs 7b and 8a), and most of them increase in abundance towards the end of the feeding phase. These polypeptides are products of the five different *R. microplus* Vgs, as observed by tBLASTn analyses (Tables 1 and 2), confirming the kinetic of Vt processing observed in previous studies^27,33^. Accordingly, these results are consistent with the current knowledge on tick female physiology, with vitellogenesis being induced by blood meal and mediated by ecdysteroids^25,34^. In *R. microplus*, adult blood feeding occurs for 7–8 days, and the last 48 hours are characterised by a rapid engorgement, when most of the blood is ingested. After completing the blood meal, the fully engorged female weight exceeds by more than 100 times that of the respective larva^3^. As the rapid blood ingestion occurs, it leads to an acceleration of the metabolism to process the meal, providing nutrients for ovary maturation and, consequently, embryogenesis^35,36^.

**Table 1:** Similarity match of vitellin poplypeptides identified in *Rhipicephalus microplus* ovary proteome, compared to *Rhipicephalus microplus* vitellogenins deposited in GenBank.

Vt ID	Best match against *Rhipicephalus microplus* Vgs^a^	Accession number
Vt_1	Vitellogenin_1	GBBR01000071.1*
	Strain Mexican vitellogenin	EU086096.1**
	GP80 precursor	U49934.1**
Vt_2	Vitellogenin_2	GBBR01000106.1*
Vt_3	Vitellogenin_2	GBBR01000106.1*
Vt_4	Vitellogenin_2	GBBR01000106.1*
Vt_5	Vitellogenin_3	GBBR01000108.1*
Vt_6	Vitellogenin_4	GBBO01000056.1*
Vt_7	Vitellogenin_5	GBBO01000066.1*

^a^Best match considering e-value 0.0.

*Transcribed RNA sequence.

**mRNA sequence, partial CDS.

**Table 2:** Similarity match of vitellin poplypeptides identified in *Rhipicephalus microplus* ovary proteome, compared to tick vitellogenins deposited in GenBank.

Vt ID	Best match against ticks Vgs^a^		Accession number
	Organism	Protein	
Vt_1	*Dermacentor variabilis*	Vitellogenin	AY885250.2**
	*Amblyomma hebraeum*	Vitellogenin 1	JX846593.1**
	*Ixodes scapularis*	Vitellogenin, putative	XM_002415179.1***
Vt_2	*Haemaphysalis longicornis*	Vitellogenin-B	AB359900.1**
	*Ixodes scapularis*	Apolipophorin, putative	XM_002401724.1***
Vt_3	*Haemaphysalis longicornis*	Vitellogenin-B	AB359900.1**
	*Ixodes scapularis*	Apolipophorin, putative	XM_002401724.1***
Vt_4	*Haemaphysalis longicornis*	Vitellogenin-B	AB359900.1**
	*Ixodes scapularis*	Apolipophorin, putative	XM_002401724.1***
Vt_5	*Dermacentor variabilis*	Vitellogenin-2	EU204907.2**
	*Amblyomma hebraeum*	Vitellogenin-2	JX846594.1**
	*Ornithodoros moubata*	Vitellogenin	AB440159.2**
	*Ixodes scapularis*	Vitellogenin, putative	XM_002403922.1***
Vt_6	*Haemaphysalis longicornis*	Vitellogenin-1	AB359899.1**
Vt_7	*Haemaphysalis longicornis*	Vitellogenin-B	AB359900.1**
	*Ixodes scapularis*	Conserved hypothetical protein	XM_002401721.1***

^a^Best match considering e-value 0.0.

**mRNA sequence, complete CDS.

***mRNA sequence.

RmVgR (*R. microplus* vitellogenin receptor, GenBank: KY781176)^32^, belonging to the low density lipoprotein receptor (LDLR)-like family, is responsible for Vg uptake from haemolymph into the oocytes. RmVgR knockdown in *R. microplus* female weighing between 25 and 35 mg results in delayed ovary development and reduced fecundity, suggesting a role in tick reproduction. RmVgR transcripts were specifically identified in the ovaries from PEF and FEF. As transcript levels decrease when females detach from the host (PEF-270), an increase in protein abundance is observed^32^. Relative quantification using dimethyl labelling corroborates RmVgR identification in all PEF groups, with a larger amount at the end of the rapid engorgement phase (PEF-189 and PEF-270) (Fig. 7a). However, receptor presence in FEF could not be detected by NSAF, probably due to a high abundance of storage proteins (e.g. Vt)^32^.

*Boophilus* yolk pro-cathepsin (BYC, GenBank: AY966003) is an acidic aspartic endopeptidase activated by autoproteolysis, that hydrolyses Vt and also haemoglobin^29^. This enzyme is synthesised in fat body and midgut and then secreted into haemolymph to be incorporated by oocytes, being allocated in the periphery of laid eggs^37^ BYC has a high specificity and low activity upon Vt, suggesting a role in continuously providing amino acids and energy for embryo development until larvae find a host^28,29^. Through dimethyl labelling relative quantification, BYC was identified in ovary from all groups between PEF-53 and PEF-270 (Fig. 7a). Also, as expected for an enzyme with a role in yolk degradation, BYC is present at higher levels in ovaries at late stages of oogenesis (PEF-270 group).

Another aspartic proteinase of which the presence in *R. microplus* ovary was confirmed is tick haeme-binding aspartic proteinase (THAP, GenBank: AF286865). This enzyme is also involved in Vt and haemoglobin hydrolysis. It is distributed in the oocyte cytoplasm, surrounding the yolk granules, and its activity is regulated by haeme^30^. THAP is synthesised in ovaries and also in fat body and midgut, being transported via haemolymph to the ovary^38^. Considering that THAP is observed as two polypeptides in FEF ovaries and in laid eggs, and that the most part of the polypeptides is converted to active enzyme 7 days after oviposition, it is conceivable that enzyme activation occurs during acidification of the yolk granules^38^. Yolk degradation is a long process, since *R. microplus* vitellogenesis takes 21 days, and a way to control THAP activity is through the haeme released by Vt proteolysis. Haeme competes with Vt to bind THAP, a negative feedback that slows down Vt degradation and prevents the oxidative damage caused by excess of haeme^30^. THAP is present in ovaries from PEF-35 to PEF-270 groups, and its abundance increases once females reach 84 mg (PEF-84 group); THAP levels keep rising until females reach 270 mg, when the feeding period is almost finished (PEF-270 group) (Fig. 7a).

Vitellin-degrading cysteine endopeptidase (VTDCE, GenBank: JQ080269) is a cathepsin L-like that hydrolyses Vt and haemoglobin^31^, and is found tightly bound to Vt in *R. microplus* eggs^39^. VTDCE is localised in midgut basal lamina and basophilic cells indicating that, just like BYC and THAP, it is synthesised in the midgut and then transported through the haemolymph to the ovaries, where it is internalised into pedicel cells and oocyte cytosol. A VTDCE inhibitor is present in female haemolymph which acts in a dose-dependent manner and is believed to control Vt hydrolysis before it is internalised into oocytes^39^. In this work, VTDCE was observed at low abundance in ovaries during the earliest phase of blood feeding, and at a higher abundance during rapid engorgement (PEF-189 and PEF-270), similarly to BYC and THAP (Fig. 7a). Also, this enzyme was detected in FEF-D2, -D3 and -D4 groups, when ovary protein extracts were probed with anti-VTDCE polyclonal antibodies (Fig. 8b).

A second cathepsin L-like that hydrolyses Vt^5,40^ and haemoglobin^5^, *Boophilus microplus* cathepsin-L like (BmCL1, GenBank: AF227957)^41^, was detected in the present analysis. This enzyme, also known as RmLCE (*Rhipicephalus microplus* larval cysteine endopeptidase), is synthesised in *R. microplus* PEF^42^ and FEF^43^ midgut, and its transcription was observed in total extract of PEF, FEF and larvae^42^. BmCL1 is localised in secretory vesicles of *R. microplus* PEF midgut, and its presence was identified also in PEF and larvae (5 to 10 days) total protein extracts^42^. This is the first report of BmCL1 presence in *R. microplus* ovary, being identified during oogenesis in PEF and FEF. The highest abundance of BmCL1 was observed during rapid engorgement (PEF-189 and PEF-270) and in the first day after detachment (FEF-D1) (Fig. S1), a profile similar to what is observed for other enzymes involved in Vt degradation. This suggests that BmCL1 is transported through haemolymph to the ovaries, and has a Vt-degrading activity mainly during embryogenesis and larval stages, as shown by Estrela *et al*.^5^.

A similar pattern of protein abundance was observed for the four enzymes involved in Vt hydrolysis (BYC, THAP, VTDCE, and BmCL1) monitored in this study. These endopeptidases accumulate in ovaries during oogenesis, and have a physiological importance at a later stage of embryo development, when they participate in yolk degradation, supplying amino acids for the synthesis of new proteins, and as a source of energy. A more gradual change was observed for the main yolk reserve protein, Vt (substrate of BYC, VTDCE, THAP, and BmCL1) (Fig. 7b), showing that during early oogenesis, Vt degradation provides the major substrate for embryo development.

### Metabolic changes during tick ovary development

Previous studies have identified a number of proteins that are involved in specific processes within oogenesis and embryogenesis in different arthropods^44–47^. Present results show that during *R. microplus* ovary development, vitellogenic proteins are upregulated from late-vitellogenic follicles to mature oocytes (i.e. from PEF-53 to more mature ovary). Meanwhile, throughout the vitellogenic process, RmVgR abundance is upregulated from early-vitellogenic oocytes (i.e., before Vt internalisation, more immature than PEF-53) to late-vitellogenic oocytes (Fig. 7a). Accordingly, different Vt peptides are observed in association with changes in abundance during the same period (Fig. 7b). These results indicate that ovary development and the consequent oocyte maturation processes occur in this tick similarly to other arthropods^48–50^. At the same time, a large variation was observed in the levels of proteins involved in carbohydrate metabolism during ovary maturation, especially enzymes involved in glycogen metabolism (e.g. glycogenin glucosyltransferase) (Fig. S2a). This is consistent with the dynamic of the final stages of oocyte maturation, which requires different compounds such as fatty acids, amino acids, electrolytes, purines and pyrimidines, and other metabolites^49^. Regarding metabolites, glucose, glycogen, lipids, and pyruvate were also observed by many authors in different arthropods^51–53^. Furthermore, carbohydrates facilitate oocyte maturation and developmental competence after fertilisation in mammals^49,54^, and are important for embryonic development in arthropods^55^, suggesting a highly conserved link between carbohydrate metabolism and oocyte development.

**Figure 9:**
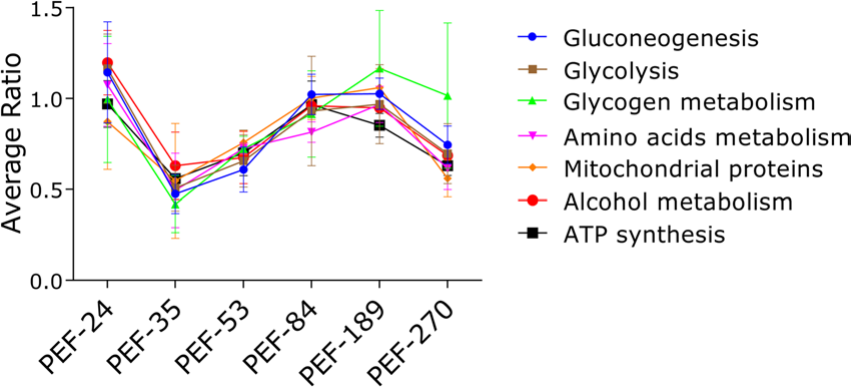
Quantification of proteins in selected metabolic pathways related to vitellogenesis. Dimethyl labelling quantification was performed in ovary extracts of partially engorged female (PEF) groups -24, -35, -53, -84, 189, -270. The ratio is the intensity detected for each peptide, relative to the internal standard (a protein pool of all ovary groups used in this study). The average ratio of a protein is calculated as the mean ratio of its peptides. The average ratio of each metabolic pathway presented in this graph was calculated from the average quantification (with standard deviation) of enzymes involved in each pathway (Supplementary Fig. S1). Numbers of ticks composing each group are: PEF-10 (n = 10), PEF-16 (n = 9), PEF-24 (n = 28), PEF-35 (n = 23), PEF-53 (n = 8), PEF-84 (n = 9), PEF-189 (n = 5), PEF-270 (n = 5).

Large differences in protein levels were identified in early-vitellogenic ovaries, indicating that maternal sources sustain egg formation, which accumulates Vt (Fig. 2). The highest levels of the studied peptidases (BYC, VTDCE, THAP, and BmCL1) are observed at the end of the vitellogenic process, simultaneously to oocyte growth. In general, proteins at low concentrations during early vitellogenesis were linked to protein processing, sugar metabolism, amino acid metabolism, mitochondrial proteins, alcohol metabolism, and ATP synthesis (Figs 9 and S2). Interestingly, the amount of these proteins increases after Vt internalisation, reflecting the energy required by this process (Figs 7b and 9). Accordingly, five proteins were found with a role in glycogen degradation (Fig. S2a), indicating this energy source is important in two critical phases of oocytes formation: in early vitellogenesis and during Vt internalisation processes. Classically, many authors have described the accumulation of glycogen during oocyte formation in different arthropods^56–58^. Likely, these mechanisms indicate that there is a metabolic energy compensation to be maintained during egg production. Furthermore, 13 proteins were found involved in glycolysis and mitochondrial metabolism (Fig. S2b and c), and 11 proteins involved in gluconeogenesis and amino acid metabolism (Fig. S2d and e), showing the importance of these metabolic pathways during egg formation and oogenesis.

## Conclusions

This work describes 3,756 proteins involved on arthropod ovary development, and evaluates the modulation of several of them. Also, the profile of proteins involved in Vt degradation was corroborated by western blot analysis. In this proteomic study, we identified a subset of proteins that have regulated abundance in ovary during ovary maturation, and their function and physiological significance are discussed. In summary, a variety of tick proteins studied as targets for the development of control methods, as well as new putative targets, were identified at different developmental stages.

## Materials and Methods

### Ethics statement

Cattle used in this work were housed at Faculdade de Veterinária, Universidade Federal do Rio Grande do Sul (UFRGS), Porto Alegre, Brazil. This research was conducted according to the ethic and methodological aspects prescribed by the International and National Directives and Norms by the Animal Experimentation Ethics Committee of UFRGS. The protocols were approved by Comissão de Ética no Uso de Animais - CEUA–Universidade Federal do Rio Grande do Sul.

### Ticks

*Rhipicephalus microplus* of Porto Alegre strain (Brazil) was reared on Hereford calves (*Bos taurus taurus*), which were brought from a naturally tick-free area and maintained in insulated pens. Calves were infested with 15-day-old tick larvae and after 22 days, partially and fully engorged adult female ticks were manually collected for ovary dissection.

### Ovary extracts and protein digestion

Partially engorged females (PEF) were manually collected from two different calves, grouped by weight in order to represent different periods of development, and then the ovaries were dissected. The weight average, SD and number of ticks in each group was as follows: 10 ± 1.73 mg (PEF-10, n = 10), 16 ± 1.22 mg (PEF-16, n = 9), 24 ± 1.90 mg (PEF-24, n = 28), 35 ± 2.84 mg (PEF-35, n = 23), 53 ± 2.49 mg (PEF-53, n = 8), 84 ± 8.36 mg (PEF-84, n = 9), 189 ± 17.17 mg (PEF-189, n = 5) and 270 ± 16.71 mg (PEF-270, n = 5). Fully engorged females (FEF) were placed in four groups according to days post-detachment. All of them were maintained at 28 °C and 85% relative humidity. Ovaries from FEF one, two, three and four days after detachment (D1, D2, D3, and D4, n = 5 for each group) were obtained by dissection. The variation in number of ticks per group was consequence of ovary size, and each sample was normalised by total protein amount. Biological variability within the samples is guaranteed by the collection of ticks made in two different calves and the number of ticks composing each group. These are the same groups used in other experiments carried on concomitantly^32^, although data was analysed independently.

Proteins from samples of each experimental group were extracted using TRIzol^®^ reagent (Thermo Fisher Scientific) following the manufacturer’s recommendations. Protein concentration was measured using BCA Protein Assay Reagent Kit, following the manufacturer’s recommendations. Ticks used for proteomic analysis were from groups PEF-10, -24, -35, -53, -84, -189 and -270, and FEF-D1 and -D3.

Methanol/chloroform protocol was employed for protein precipitation^50^. Dried pellets were dissolved in 8 M urea/100 mM Tris, pH 8.5. Proteins were reduced with 5 mM tris(2-carboxyethyl)phosphine hydrochloride (TCEP, Sigma-Aldrich) and alkylated with 25 mM iodoacetamide (Sigma-Aldrich). Proteins were hydrolysed overnight at 37 °C in 2 M urea/100 mM Tris pH 8.5, 1 mM CaCl_2_ with trypsin (Promega) in a final ratio of 1:100 (enzyme:protein). Digestion was stopped by adding formic acid (5% final concentration). Debris were removed by centrifugation. For quantitative experiments using dimethyl labelling, hydrolysis reactions were buffered with 100 mM tetraethylammonium bromide (TEAB), pH 8.5, instead of Tris, and samples were kept at −80 °C until use, without acidification.

### Dimethyl labelling

Dimethyl labelling was performed according to Boersema *et al*.^59^. Samples (30 µg) dissolved in 2 M urea/100 mM TEAB were diluted with 100 mM TEAB to a final volume of 100 µL. Dimethyl labelling reactions were initiated by adding 8 µL of a 4% v/v formaldehyde solution and 8 µL of 0.6 M sodium cyanoborohydride, vortexed and incubated for 1 hour at room temperature. Reactions were stopped by addition of 8 µL of 1 M NH_4_HCO_3_ and further quenched by acidification with 8 µL of 95% formic acid. Isotopic labelling (light, medium and heavy) was performed by combining isotopic forms of formaldehyde and sodium cyanoborrohydride as follows: CH_2_O + NaBH_3_CN for light; CD_2_O + NaBH_3_CN for intermediate, ^13^CD_2_O + NaBD_3_CN for heavy. Peptides are labelled at free amines (N-terminus and lysine side chain), resulting in mass shifts of +28.0313 (light), +32.0564 (medium) and +36.0757 (heavy) per label incorporated.

Samples PEF-24, -53 and -189 were labelled with intermediate dimethylation; samples PEF-35, -84 and -270 were labelled with heavy dimethylation. Light dimethylation was used to label a pool of equal amounts of all six samples, to generate an internal standard. When combined for the quantitative analysis, pairs of intermediate and heavy samples were analysed in the presence of this light internal standard (standard:PEF-24:PEF-35, standard:PEF-53:PEF-84 and standard:PEF-189:PEF-270), allowing cross comparison between samples.

### MuDPIT and analytical columns

MuDPIT (Multidimensional Protein Identification Technology) columns were prepared by first creating a Kasil frit at one end of a deactivated 250 µm ID/360 µm OD capillary (Agilent Technologies). Kasil frits were prepared by dipping 20 cm capillary in 300 µL Kasil 1624 (PQ Corporation) and 100 µL formamide solution, curing at 100 °C for 3 hours, and cutting the frit to a length of ~2 mm. A biphasic MuDPIT column was produced in the fritted column by packing in-house 2.5 cm of a strong cation exchange (SCX) resin (5 µm Partisphere, Phenomenex), followed by 2.5 cm of reverse phase resin (5 µm ODS-AQ C18, YMC); both resins slurries were stored in methanol. Analytical reverse phase columns were prepared by pulling a 100 µm ID/360 µm OD capillary (Polymicro Technologies) into a 5 µm ID tip. Reverse phase resin (5 µm ODS-AQ C18, YMC) was packed directly into the pulled column until 18 cm in length. MuDPIT columns and analytical columns were connected using a zero-dead volume union (Upchurch Scientific).

### LC-MS/MS

Peptide mixtures were analysed by LC-MS using quaternary HP 1100 series HPLC (high performance liquid chromatography) pump (Agilent technology) connected to an LTQ (Linear Trap Quadropole) XL or an LTQ Orbitrap Velos mass spectrometer (Thermo Scientific). Electrospray was performed directly from the tip of the analytical column. Mobile phases were as follows: solution A, 5% acetonitrile and 0.1% formic acid; solution B, 80% acetonitrile and 0.1% formic acid; solution C, 500 mM ammonium acetate, 5% acetonitrile and 0.1% formic acid. Flow rate was approximately 300 nL/min.

MuDPIT experiments were performed using a 45-minute transfer step followed by ten 2-hour cycles of salt pulse (solution C injection) and separation steps. Transfer step consisted of a linear gradient from 0 to 60% B over 25 minutes, followed by an increase to 100% B in 5 minutes. Column was re-equilibrated with 100% A for 10 minutes. Salt pulse consisted of a 4-minute solution C injection followed by 6 minutes of 100% A. Peptide separation gradient was: from 0 to 10% B in 5 minutes, increase to 20% B over 35 minutes, increase to 50% B over 40 minutes, and increase to 100% B in 15 minutes. Column was flushed at 100% B for 5 minutes and re-equilibrated in 100% A for 20 minutes. At each of the ten cycles, mobile phase during the salt pulse consisted of 10, 20, 30, 40, 50, 60, 70, 85, or 100% C, or 90% C/10% B. Due to a slight increase in peptide hydrophobicity, for dimethyl labelling experiments, the cycle with 40% C was omitted, and an extra cycle was performed in the end with 90% C/10% B.

The LTQ XL was operated in a data dependent scan mode, with ESI voltage of 3 kV and inlet capillary temperature of 275 °C. Full MS1 scans and CID MS2 scans of the 5 most abundant ions in each cycle were collected on the LTQ ion trap. AGC target was 3e^4^ and 1e^4^ for MS1 and MS2, respectively, and the mass range was 300 to 1200 m/z. Maximum injection times were 50 ms for MS1 and 100 ms for MS2 scans. Dynamic exclusion was enabled with repeat count of 1, repeat duration of 30 s, exclusion list size of 50 and exclusion duration of 30 s.

The LTQ Orbitrap Velos was also operated in a data-dependent mode, ESI voltage of 3.5 kV and inlet capillary temperature of 275 °C. Full MS1 scans were collected in the Orbitrap, with mass range of 300 to 1200 m/z at 60 K resolution and an AGC target of 1e^6^. The 20 most abundant ions per MS1 scan were selected for CID MS2 in the LTQ Velos ion trap, with an AGC target of 1e^4^ and threshold intensity of 500. Maximum fill times were 250 ms and 100 ms for MS1 and MS2 scans, respectively, and dynamic exclusion was used with repeat count of 1, repeat duration of 150 s, exclusion list size of 500 and exclusion duration of 120 s.

### LC-MS/MS data analysis

Label-free quantitative analysis was performed by comparing single MuDPIT technical replicates of each tick ovary groups (PEF-10, -24, -35, -53, -84, -189 and -270, and FEF-D1 and -D3). However, as the incomplete elution of peptides from ion exchange column allows peptide identification in more than one fraction, these fractions are analysed together, generating a unique result with redundant peptide data. The semi-quantitative method based on NSAF (normalised spectral abundance factor) was used. NSAF for a given protein is the number of spectral counts (SpC) identified for that protein, divided by the protein’s length (L), divided by the sum of SpC/L of all protein in the experiment.

Dimethyl labelling quantitative analysis was performed in ovary groups labelled as described above (section Dimethyl labelling). One MuDPIT technical replicate for each sample pair and internal standard (standard:PEF-24:PEF-35, standard:PEF-53:PEF-84 and standard:PEF-189:PEF-270) was used. Due to MuDPIT characteristics, one peptide can be identified and quantified in more than one fraction in the same run. This redundancy of measurements supports data reliability.

Protein and peptide identification was done with Integrated Proteomics Pipeline–IP2 (Integrated Proteomics Applications). Tandem mass spectra were extracted from raw files using RawExtract 1.9.9.2^60^ and searched with ProLuCID^61^ against a local *R. microplus* protein database (Rm-INCT-EM) containing 22,010 sequences previously produced by our research group using Illumina Sequencing technology (BioProject ID PRJNA232001 at Transcriptome Shotgun Assembly (TSA) database–GenBank). The search space included all fully-tryptic and half-tryptic peptide candidates. Carbamidomethylation on cysteine residues (+57.02146) was used as a static modification. For dimethyl labelling, dimethylation (+28.0313) of N-terminus and lysines was considered as static modification, and the mass difference between intermediate and light labels (+4.0251), or between heavy and light labels (+8.0444), were considered as metabolic labelling. Data was searched with 50 ppm precursor ion tolerance and 600 ppm fragment ion tolerance. Identified proteins were filtered using DTASelect^62^. Filtering required a minimum of 2 peptides per protein, at least one tryptic terminus for each peptide, and less than 1% FDR. Normalised spectral abundance factor (NSAF) was calculated according to Zybailov *et al*.^63^. Comparisons between runs were performed by identificationCOMPARE, part of the IP2 pipeline. Quantitative analysis was performed by Census integrated into IP2 pipeline^64^. Protein ratios (medium/light and heavy/light) were calculated by quantitativeCOMPARE, using peptide ratios and including redundant proteins.

### Protein functional annotation

The functional annotation and clusterisation of protein sequences were performed using Visual Basic programs developed by Dr. José Marcos Ribeiro^65^. BLASTP tool was used to search proteins sequences against several databases. The final result presented here was manually curated and, in many cases, manually annotated. This catalogue was plotted on a hyperlinked Microsoft Excel^®^ spreadsheet (see Supplementary Table S1).

The abundance profile of dimethyl-labelled proteins was analysed by normalizing each PEF group relative to PEF-24 group. Proteins with *p* value and ratio variance ≥0.06 were excluded from the analysis (see Supplementary Table S3).

### Relative abundance and graphical visualisation

Proteomic profiles were compared across samples as functional clusters of proteins. To determine the relative abundance of proteins, NSAF was used in a label-free relative quantification approach^66^. NSAF as an index for relative protein abundance was input in Microsoft Excel^®^ as percentage of the total NSAF for respective samples, and visualised on pie charts according to protein clusters. To visualise relative protein patterns on a heat map, NSAF values were normalised using Z-score. Normalised NSAF values were used to generate heat maps using the heatmap2 function from the gplots library in R.

### SDS-PAGE and Western Blot

Tick ovary protein extracts were analysed by 10% SDS-PAGE (sodium dodecyl sulphate–polyacrylamide gel electrophoresis). The gel was loaded with 50 µg of protein per sample lane (PEF-10, -16, -24, -35, -53, -84, -189 and -270; and FEF-D1, -D2, -D3 and -D4) and stained with coomassie brilliant blue for protein visualisation.

The presence of vitellin and VTDCE in ovary extracts was evaluated by western blot. Fifty micrograms of each tissue extract were resolved by SDS-PAGE and eletroblotted on nitrocellulose membranes. Membranes were blocked with 5% non-fat dry milk in PBS (blocking solution), for 1 hour at room temperature. Membranes were incubated with anti-vitellin^33^ or anti-VTDCE^67^ rabbit sera diluted 1:100 and 1:200, respectively, in blocking solution for 16 h at 4 °C. After three washes with blocking solution, membranes were incubated with anti-rabbit antibodies conjugated with alkaline phosphatase (diluted 1:5,000 in blocking solution) for 1 hour at room temperature. After washes with PBS and alkaline phosphatase buffer (Tris-HCl 100 mM, NaCl 100 mM and MgCl_2_ 5 mM), membranes were incubated with NBT (nitro blue tetrazolium, Thermo Scientific; 0.3 mg/mL) and BCIP (5-bromo-4-chloro-3-indolyl phosphate, Fermentas; 0.15 mg/mL) in alkaline phosphatase buffer.

## Electronic supplementary material

Supplementary Information

Table S1

Table S2

Table S3

## References

[1] Dantas-Torres F, Chomel BB, Otranto D (2012). Ticks and tick-borne diseases: a one health perspective. Trends in Parasitology.

[2] Grisi L (2014). Reassessment of the potential economic impact of cattle parasites in Brazil. Braz. J. Vet. Parasitol., Jaboticabal.

[3] Roberts JA (1968). Resistance of cattle to the tick *Boophilus microplus* (canestrini). II. Stages of the life cycle of the parasite against which resistance is manifest. J. Parasitol..

[4] Oliver JH (1989). Biology and systematics of ticks (Acari:Ixodida). Annu. Rev. Ecol. Syst..

[5] Estrela AB, Seixas A, Teixeira VDON, Pinto AFM, Termignoni C (2010). Vitellin- and hemoglobin-digesting enzymes in *Rhipicephalus (Boophilus) microplus* larvae and females. Comp. Biochem. Physiol..

[6] Rosell R, Coons LB (1992). The role of the fat body, midgut and ovary in vitellogenin production and vitellogenesis in the female tick. Dermacentor variabilis. Int. J. Parasitol..

[7] Thompson DM (2007). Sequence and the developmental and tissue-specific regulation of the first complete vitellogenin messenger RNA from ticks responsible for heme sequestration. Insect Biochem. Mol. Biol..

[8] Boldbaatar D (2010). Multiple vitellogenins from the *Haemaphysalis longicornis* tick are crucial for ovarian development. J. Insect Physiol..

[9] Khalil SMS (2011). Full-length sequence, regulation and developmental studies of a second vitellogenin gene from the American dog tick, *Dermacentor variabilis*. J. Insect Physiol..

[10] Giorgi F, Bradley JT, Nordin JH (1999). Differential vitellin polypeptide processing in insect embryos. Micron.

[11] Raikhel AS, Dhadialla TS (1992). Accumulation of yolk proteins in insect oocytes. Annu. Rev. Entomol..

[12] Oppenheim SJ, Baker RH, Simon S, Desalle R (2015). We can’t all be supermodels: the value of comparative transcriptomics to the study of non-model insects. Insect Molecular Biology.

[13] Ewen-Campen B (2011). The maternal and early embryonic transcriptome of the milkweed bug *Oncopeltus fasciatus*. BMC Genomics.

[14] Gokhale K (2013). Transcriptome analysis of *Anopheles stephensi* embryo using expressed sequence tags. J. Biosci..

[15] Seixas A (2012). *Rhipicephalus (Boophilus) microplus* embryo proteins as target for tick vaccine. Vet. Immunol. Immunopathol..

[16] Mateo Leach I (2009). Transcriptome and proteome analysis of ovaries of arrhenotokous and thelytokous *Venturia canescens*. Insect Mol. Biol..

[17] Cardoen D (2012). Worker honeybee sterility: a proteomic analysis of suppressed ovary activation. J. Proteome Res..

[18] Cui J, Wu LT, Chu KH (2014). Comparative proteomic profiling during ovarian development of the shrimp *Metapenaeus ensis*. Mol. Biol. Rep..

[19] Talakhun W (2014). Proteomic analysis of ovarian proteins and characterization of thymosin-β and RAC-GTPase activating protein 1 of the giant tiger shrimp *Penaeus monodon*. Comp. Biochem. Physiol. - Part D Genomics Proteomics.

[20] Santos VT (2013). The embryogenesis of the tick *Rhipicephalus (Boophilus) microplus*: the establishment of a new chelicerate model system. Genesis.

[21] Rachinsky A, Guerrero FD, Scoles GA (2007). Differential protein expression in ovaries of uninfected and *Babesia*-infected southern cattle ticks, *Rhipicephalus (Boophilus) microplus*. Insect Biochem. Mol. Biol..

[22] Heekin AM (2013). The ovarian transcriptome of the cattle tick, *Rhipicephalus (Boophilus) microplus*, feeding upon a bovine host infected with *Babesia bovis*. Parasit. Vectors.

[23] Balashov YS (1972). Bloodsucking ticks (Ixodoidea) — vectors of diseases of man and animals. Misc. Publ. Entomol. Soc. Am..

[24] Chinzei Y, Okuda T, Ando K (1989). Vitellogenin synthesis and ovarian development in nymphal and newly molted female *Ornithodoros moubata* (Acari: Argasidae). J. Med. Entomol..

[25] Seixas A, Friesen KJ, Kaufman WR (2008). Effect of 20-hydroxyecdysone and haemolymph on oogenesis in the ixodid tick *Amblyomma hebraeum*. J. Insect Physiol..

[26] Velentzas AD (2015). Global proteomic profiling of *Drosophila* ovary: a high-resolution, unbiased, accurate and multifaceted analysis. Cancer Genomics and Proteomics.

[27] Logullo C (2002). Binding and storage of heme by vitellin from the cattle tick. Boophilus microplus. Insect Biochem. Mol. Biol..

[28] Nascimento-Silva MCL (2008). BYC, an atypical aspartic endopeptidase from *Rhipicephalus (Boophilus) microplus* eggs. Comp. Biochem. Physiol. - B Biochem. Mol. Biol..

[29] Logullo C (1998). Isolation of an aspartic proteinase precursor from the egg of a hard tick. Boophilus microplus. Parasitology.

[30] Sorgine MHF (2000). A heme-binding aspartic proteinase from the eggs of the hard tick *Boophilus microplus*. J. Biol. Chem..

[31] Seixas A (2003). A *Boophilus microplus* vitellin-degrading cysteine endopeptidase. Parasitology.

[32] Seixas A (2018). Expression profile of *Rhipicephalus microplus* vitellogenin receptor during oogenesis. Ticks Tick. Borne. Dis..

[33] Canal CW (1995). Changing patterns of vitellin-related peptides during development of the cattle tick *Boophilus microplus*. Exp. Appl. Acarol..

[34] Friesen KJ, Reuben Kaufman W (2002). Quantification of vitellogenesis and its control by 20-hydroxyecdysone in the ixodid tick, *Amblyomma hebraeum*. J. Insect Physiol..

[35] Uspensky I, Ioffe-Uspensky I (1999). The relationship between engorged female weight and egg number in ixodid ticks: a biological interpretation of linear regression parameters. Acarologia.

[36] Hao J (2017). MicroRNA-275 and its target Vitellogenin-2 are crucial in ovary development and blood digestion of *Haemaphysalis longicornis*. Parasit. Vectors.

[37] Abreu LA (2004). Proteolytic activity of *Boophilus microplus* Yolk pro-Cathepsin D (BYC) is coincident with cortical acidification during embryogenesis. Insect Biochem. Mol. Biol..

[38] Pohl PC (2008). An extraovarian aspartic protease accumulated in tick oocytes with vitellin-degradation activity. Comp. Biochem. Physiol. - B Biochem. Mol. Biol..

[39] Seixas A (2010). Localization and function of *Rhipicephalus (Boophilus) microplus* vitellin-degrading cysteine endopeptidase. Parasitology.

[40] Estrela A, Seixas A, Termignoni C (2007). A cysteine endopeptidase from tick (*Rhipicephalus (Boophilus) microplus*) larvae with vitellin digestion activity. Comp. Biochem. Physiol. Part B Biochem. Mol. Biol..

[41] Renard G (2000). Cloning and functional expression of a *Boophilus microplus* cathepsin L-like enzyme. Insect Biochem. Mol. Biol..

[42] Renard G (2002). Expression and immunolocalization of a *Boophilus microplus* cathepsin L-like enzyme. Insect Mol. Biol..

[43] Clara RO (2011). *Boophilus microplus* cathepsin L-like (BmCL1) cysteine protease: specificity study using a peptide phage display library. Vet. Parasitol..

[44] Roth TF, Porter KR (1964). Yolk protein uptake in the oocyte of the mosquito *Aedes aegypti*. L. J. Cell Biol..

[45] Fagotto F (1990). Yolk degradation in tick eggs: I. Occurrence of a cathepsin L‐like acid proteinase in yolk spheres. Arch. Insect Biochem. Physiol..

[46] Sappington TW, Raikhel S (1998). A. Molecular characteristics of insect vitellogenins and vitellogenin receptors. Insect Biochemistry and Molecular Biology.

[47] Carter J-M (2013). Unscrambling butterfly oogenesis. BMC Genomics.

[48] Moraes J (2007). Glucose metabolism during embryogenesis of the hard tick *Boophilus microplus*. Comp. Biochem. Physiol. - A Mol. Integr. Physiol..

[49] Lim JM (1999). *In vitro* maturation and *in vitro* fertilization of bovine oocytes cultured in a chemically defined, protein-free medium: effects of carbohydrates and amino acids. Reprod Fertil Dev.

[50] Wessel D, Flügge UI (1984). A method for the quantitative recovery of protein in dilute solution in the presence of detergents and lipids. Anal. Biochem..

[51] Vital W (2010). Germ band retraction as a landmark in glucose metabolism during *Aedes aegypti* embryogenesis. BMC Dev. Biol..

[52] Fraga, A. *et al*. Glycogen and glucose metabolism are essential for early embryonic development of the red flour beetle *Tribolium castaneum*. *PLoS ONE* **8** (2013). 10.1371/journal.pone.0065125 PMC3672164 23750237

[53] Zeng Z, Ni J, Ke C (2015). Glycogen content relative to expression of glycogen phosphorylase (GPH) and hexokinase (HK) during the reproductive cycle in the Fujian Oyster. Crassostrea angulata. Acta Oceanol. Sin..

[54] Rooke JA (2009). Dietary carbohydrates and amino acids influence oocyte quality in dairy heifers. Reprod. Fertil. Dev..

[55] Atella GC (2005). Oogenesis and egg development in triatomines: a biochemical approach. An. Acad. Bras. Cienc..

[56] Yamazaki H, Yanagawa SI (2003). Axin and the Axin/Arrow-binding protein DCAP mediate glucose-glycogen metabolism. Biochem. Biophys. Res. Commun..

[57] Abreu LAde (2009). Exogenous insulin stimulates glycogen accumulation in *Rhipicephalus (Boophilus) microplus* embryo cell line BME26 via PI3K/AKT pathway. Comp. Biochem. Physiol. - B Biochem. Mol. Biol..

[58] Sieber MH, Thomsen MB, Spradling AC (2016). Electron transport chain remodeling by GSK3 during oogenesis connects nutrient state to reproduction. Cell.

[59] Boersema PJ, Raijmakers R, Lemeer S, Mohammed S, Heck AJR (2009). Multiplex peptide stable isotope dimethyl labeling for quantitative proteomics. Nat. Protoc..

[60] McDonald WH (2004). MS1, MS2, and SQT - Three unified, compact, and easily parsed file formats for the storage of shotgun proteomic spectra and identifications. Rapid Commun. Mass Spectrom..

[61] Xu T (2015). ProLuCID: An improved SEQUEST-like algorithm with enhanced sensitivity and specificity. J. Proteomics.

[62] Tabb DL, McDonald WH, Yates JR (2002). DTASelect and Contrast: Tools for assembling and comparing protein identifications from shotgun proteomics. J. Proteome Res..

[63] Zybailov B (2006). Statistical analysis of membrane proteome expression changes in *Saccharomyces cerevisiae*. J. Proteome Res..

[64] Park SKR (2014). Census 2: Isobaric labeling data analysis. Bioinformatics.

[65] Karim S, Singh P (2011). A deep insight into the sialotranscriptome of the gulf coast tick, *Amblyomma maculatum*. PLoS ONE.

[66] Paoletti AC (2006). Quantitative proteomic analysis of distinct mammalian mediator complexes using normalized spectral abundance factors. Proc. Natl. Acad. Sci. USA.

[67] Seixas A (2008). Vaccine potential of a tick vitellin-degrading enzyme (VTDCE). Vet. Immunol. Immunopathol..

