# Supplementary material for: A proteomic insight into vitellogenesis during tick ovary maturation
Source: Sci Rep. 2018 Mar 16;8:4698. doi: 10.1038/s41598-018-23090-2 (PMC5856802; doi:10.1038/s41598-018-23090-2)
Supplement: Supplementary file 1 — Supplementary Information [file 41598_2018_23090_MOESM1_ESM.doc]

**Supplementary Information**

**A proteomic insight into vitellogenesis during tick ovary maturation**

Marina Amaral Xavier, Lucas Tirloni, Antônio F. M. Pinto, Jolene K. Diedrich, John R. Yates III, Albert Mulenga, Carlos Logullo, Itabajara da Silva Vaz Jr., Adriana Seixas, Carlos Termignoni

**
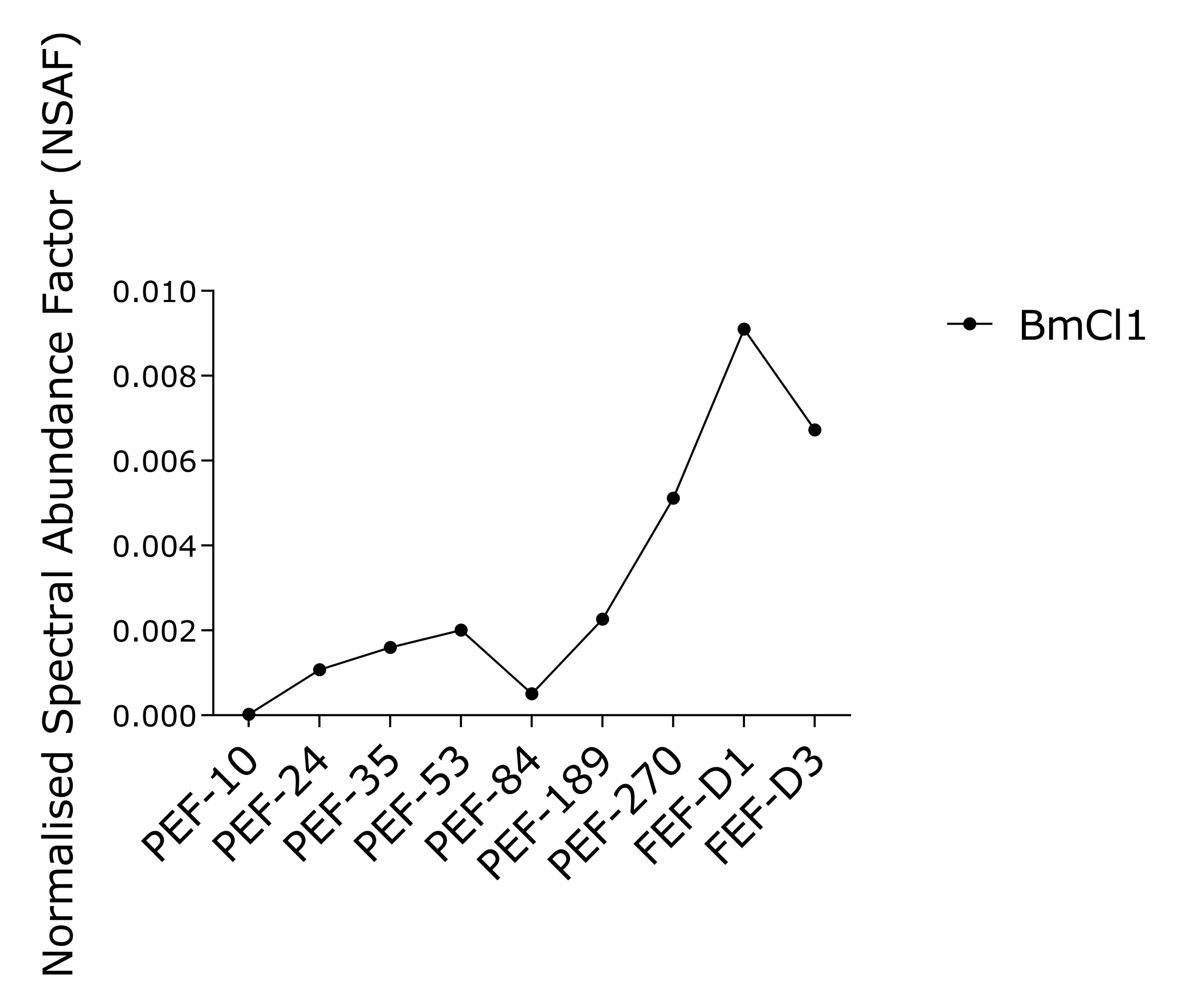
**

**Supplementary Figure S1: Profile of abundance of *Boophilus microplus* cathepsin-L like (BmCL1) in *Rhipicephalus microplus* ovaries.** Normalised spectral abundance factor (NSAF) semi-quantitative analysis was performed in ovary extracts of partially engorged female groups PEF-10, -24, -35, -53, -84, -189 and -270, and in fully engorged female groups FEF-D1 and -D3. The chart shows NSAF of BmCL1, a protease implicated in yolk degradation.


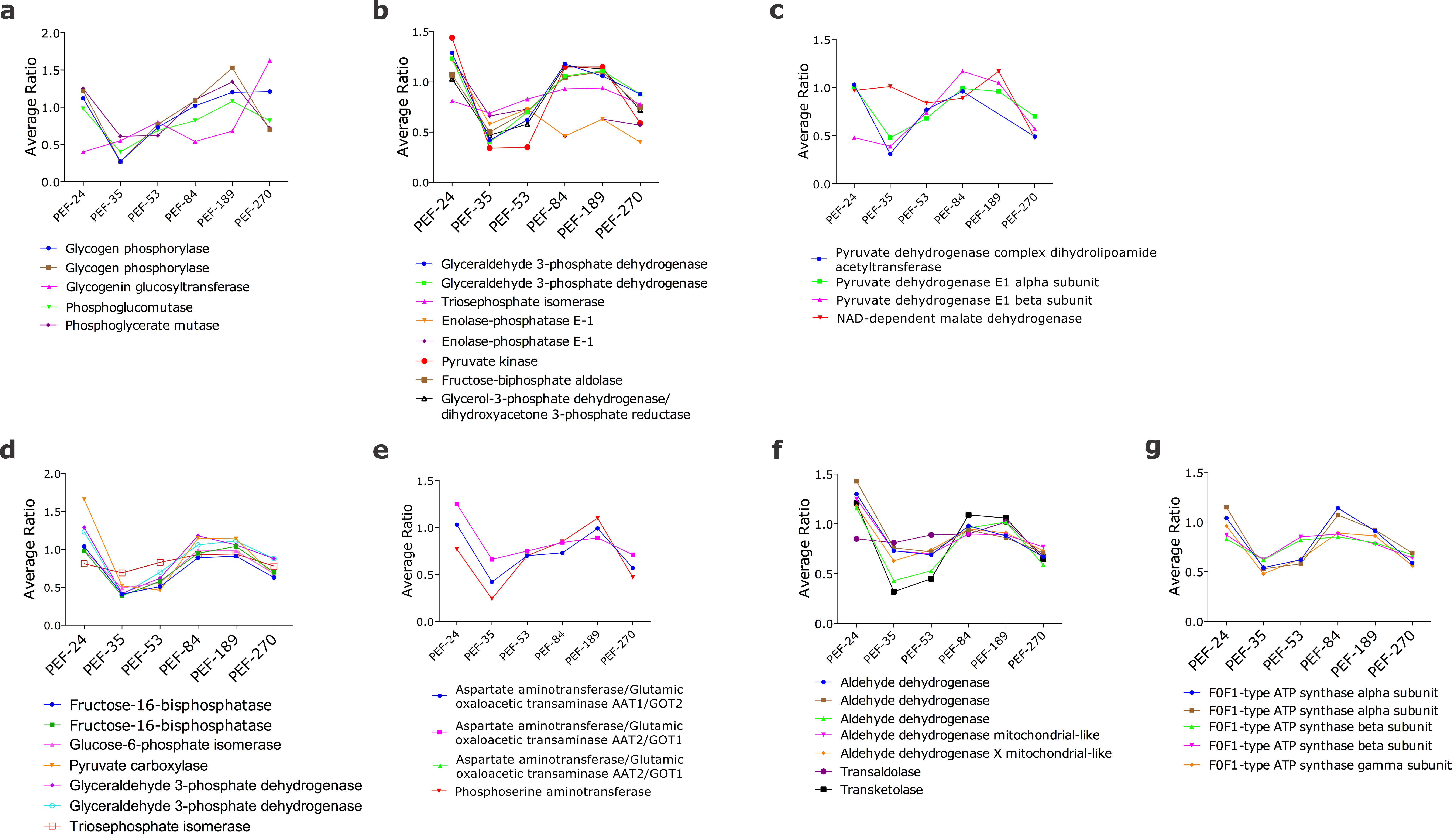


**Supplementary Figure S2: Detailed selected metabolic pathways involved in vitellogenesis.** Dimethyl labelling quantification of proteins was performed in ovary extracts of partially engorged female groups PEF-24, -35, -53, -84, 189, -270. The ratio is the intensity detected for each peptide, relative to the internal standard (a protein pool of all samples used in this study). Thus, the average ratio of a protein is calculated as the mean ratio of its peptides. Charts show protein average ratio of enzymes involved in: (a) glycogen metabolism; (b) glycolysis; (c) mitochondrial proteins; (d) gluconeogenesis; (e) amino acids metabolism; (f) alcohol metabolism; (g) ATP synthesis.

**Supplementary Table S1:** ***Rhipicephalus microplus* ovary proteins identified by mass spectrometry.** Protein annotation is based on function and/or protein family. The accession numbers and other specifications of the best match, obtained by BLASTP alignment against several databases, are described.

**Supplementary Table S2: Semi-quantitative analysis of *Rhipicephalus microplus* ovary proteins.** Identification data of proteins in each tick group, partially (PEF) and fully (FEF) engorged females, are provided: peptide count, normalised spectral abundance factor (NSAF), exponentially modified protein abundance index (emPAI), spectral count and sequence coverage.

**Supplementary Table S3: Quantitative analysis of proteins from *Rhipicephalus microplus* partially engorged females (PEF) ovaries.** Data from dimethyl labelling quantification of ovary proteins in PEF groups -24, -35, -53, -84, -189 and -270 is provided. Specifications for each tick group are in separate sheet; proteins with *p* value and ratio variance ≥ 0.06 were excluded from the analysis.
